# Efficacy of Oblique Lateral Interbody Fusion at L5/S1 for Lumbosacral Transitional Vertebrae-Related Far-Out Syndrome: A Report of Two Cases

**DOI:** 10.7759/cureus.79431

**Published:** 2025-02-21

**Authors:** Yoshinori Maki, Kenji Fukaya

**Affiliations:** 1 Department of Neurosurgery, Ayabe Renaiss Hospital, Ayabe, JPN; 2 Department of Neurosurgery, Hikone Chuo Hospital, Hikone, JPN

**Keywords:** anterior decompression, far-out syndrome, l5 radiculopathy, lumbosacral transitional vertebrae, oblique lateral interbody fusion, posterior decompression

## Abstract

Far-out syndrome is often related to lumbosacral transitional vertebrae (LSTV) and can result in L5 radiculopathy. Although conservative treatment can resolve the radiculopathy, surgical treatment should be considered when the symptoms are refractory. Several surgical procedures, including facetectomy with fusion surgery and nerve root decompression via the anterior or posterior approach, have been described. However, research regarding the efficacy of oblique lumbar intervertebral fusion at L5/S1 (OLIF51) to resolve far-out syndrome is limited. This case report aimed to report the efficacy of this surgery. OLIF51 was performed for two patients with far-out syndrome. The first patient (an 81-year-old man) had undergone posterior lumbar intervertebral fixation from L3 to L5 for degenerative lumbar spondylosis. The right refractory L5 radiculopathy persisted after surgery. As the L5 radiculopathy might have resulted from LSTV, posterior nerve decompression surgery was performed. However, the symptoms recurred after one month, and OLIF51 was performed to decompress the L5 nerve root in the anterior exit zone. The second patient (a 46-year-old man) had a disc herniation at L4/5, which was treated surgically five months prior. Because the right L5 radiculopathy related to LSTV persisted, OLIF51 was performed. Following OLIF51, the L5 nerve root radiculopathy disappeared in both cases. Both patients have been followed up at the outpatient clinic, and their symptoms have not recurred for 12 and 10 months in the first and second patients, respectively. OLIF51 can effectively resolve far-out syndrome related to LSTV.

## Introduction

Far-out syndrome, initially reported by Wiltse et al. in 1984 [1], is a rare nerve entrapment condition affecting the L5 nerve root. This syndrome can manifest symptomatically when congenital bony abnormalities at the L5/S1 level, i.e., the lumbosacral transitional vertebrae (LSTV), and degenerative changes of the bone, intervertebral discs, and ligaments cause narrowing of the extraforaminal area surrounded by the transverse process of the L5 vertebra and ala of the sacrum [2,3].

Conservative treatments, such as medications, nerve block, rest, and steroid injections, are the first-line treatments for managing far-out syndrome [2-4]. In case conservative treatment fails to control the symptoms of far-out syndrome, surgical treatment can be considered. Because of the rarity of this syndrome, a standardized surgical approach has not been established. Since the time Wiltse et al. described unilateral total facetectomy combined with lumbar fusion [1], other techniques, such as LSTV resection, laminectomy, L5 nerve root decompression via anterior approach or posterior approach, and endoscopic L5 nerve root decompression, have also been reported [2,3,5,6]. However, research regarding the efficacy of oblique lumbar intervertebral fusion at L5/S1 (OLIF51) in resolving far-out syndrome is limited. Here, we present two cases of far-out syndrome successfully treated with OLIF51.

## Case presentation

Case 1

**Figure 1:**
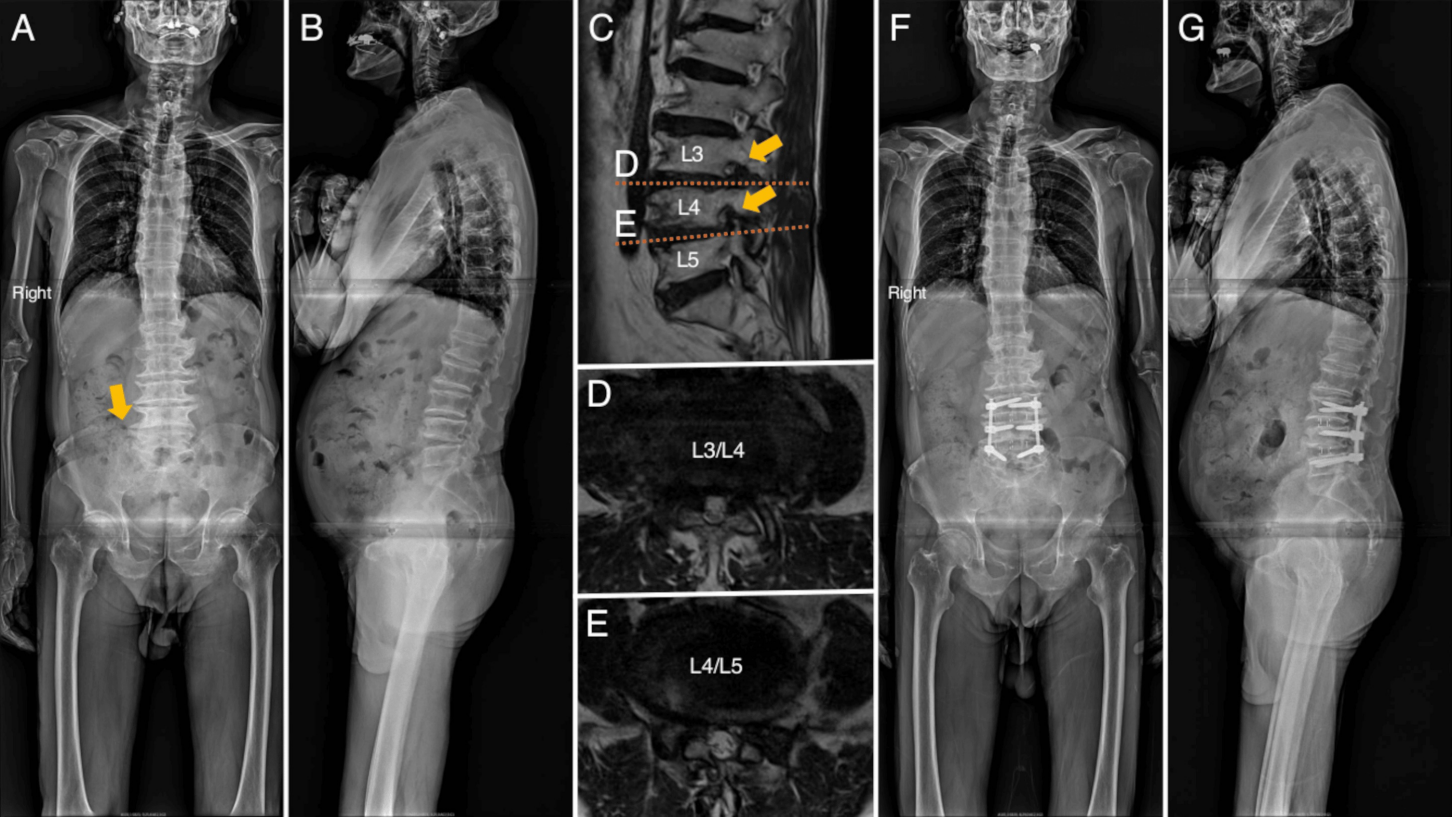
Radiological images of Case 1 before oblique lumbar intervertebral fusion at L5/S1 (A, B) The right lumbosacral transitional vertebra was retrospectively observed on X-ray images (orange arrow). (C-E) Right L3 and L4 foraminal stenosis (orange arrows) and degenerative lumbar spondylosis were observed. (F, G) X-ray images showed intervertebral discs and pedicle screws inserted in posterior fixation surgery from L3 to L5 A, F: coronal X-ray images; B, G; sagittal X-ray images; C: sagittal preoperative MRI; D, E: axial preoperative MRI

An 81-year-old man presented to our hospital with complaints of pain in the back and right lower extremity. A magnetic resonance image (MRI) examination was performed to rule out lumbar lesions. Lumbar canal stenosis from L3 to L5 and foraminal stenosis of L3 and L4 nerve roots were disclosed, and those regions seemed causative of the symptoms. Posterior fixation surgery from L3 to L5 was performed for the degenerative lumbar spondylosis. The pain disappeared temporarily after the surgery but recurred after two weeks, corresponding to the right L5 nerve root dermatome. Retrospectively, LSTV involvement was confirmed (Figure 1).

**Figure 2:**
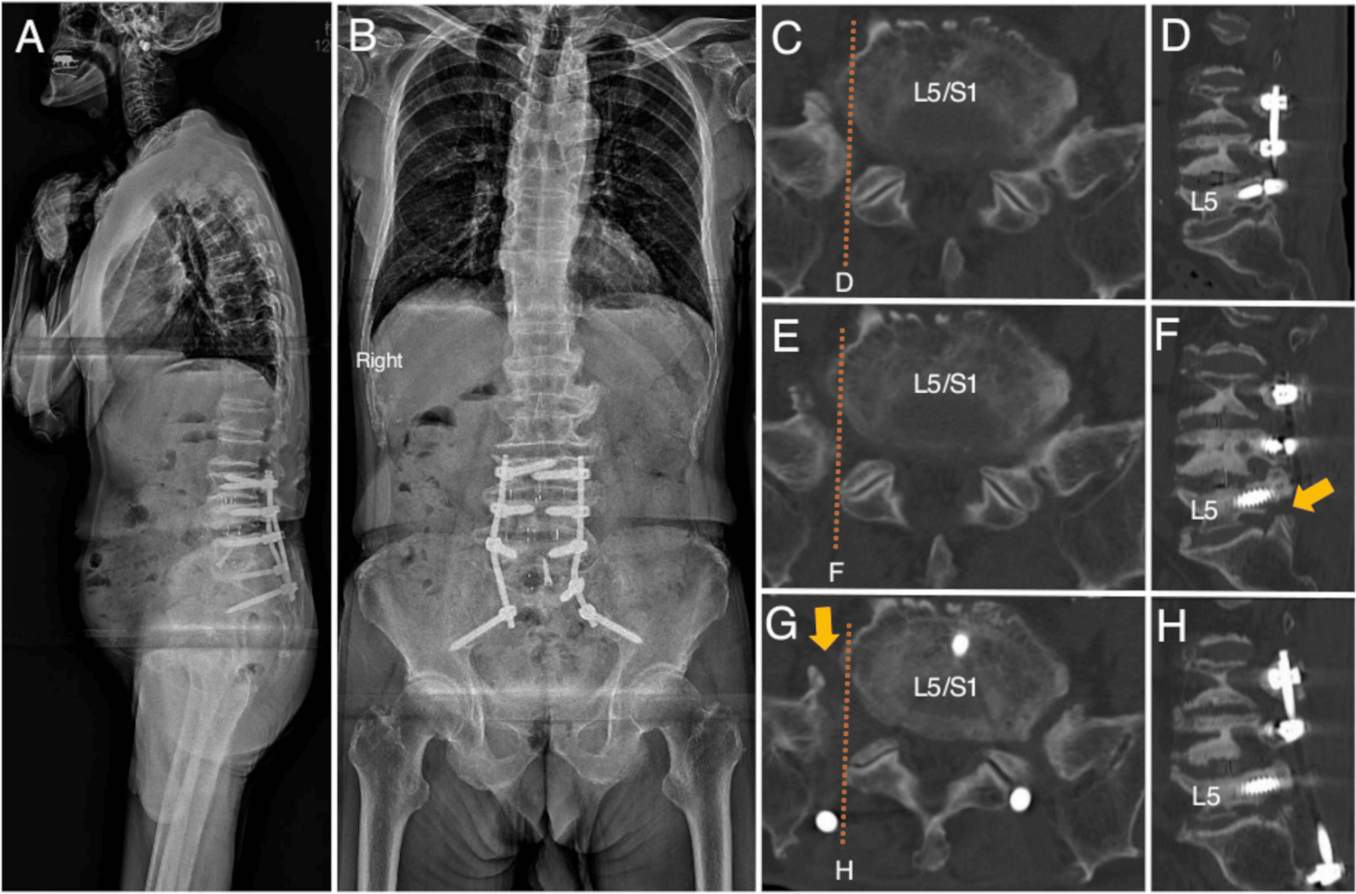
X-ray images after oblique lumbar intervertebral fusion at L5/S1 and chronological computed tomography images of Case 1. (A, B) Following oblique lumbar intervertebral fusion at L5/S1 (OLIF51), posterior fixation was extended from L3 to the ilium. The additional intervertebral disc at L5/S1 and screws at S1 and the ilium were observed on postoperative sagittal and coronal X-ray images. (C, D) The foraminal stenosis of the right L5 nerve root was observed on axial and sagittal computed tomography images. (E, F) The posterior foraminal decompression was achieved (F, orange arrow). Still, the anterior exit stenosis of the right L5 nerve root remained even after posterior decompression. (G, H) The anterior exit stenosis of the right L5 nerve root was resolved after OLIF51 (G, orange arrow). A, B: sagittal X-ray images; B, coronal X-ray image; C, E, G: axial CT images; D, F, H: sagittal CT images; C, D: before second surgery; E, F: after second surgery and before third surgery; G, H: after third surgery

Therefore, a diagnosis of far-out syndrome at the L5/S1 level was made. Since medications did not relieve the pain, posterior decompression of the L5 nerve root was performed after four months. The pain was resolved but reappeared one month after the second operation. The persistent pain seemed to be attributed to insufficient decompression of the anterior exit zone of the right L5 nerve root. Thus, OLIF51 was performed to release the right L5 nerve root anteriorly. An artificial intervertebral cage (degree: 12°, size: 32mm × 23mm × 14mm, SOVEREIGN™ Spinal System; Medtronic plc, Galway, Ireland) was inserted into the L5/S1 intervertebral space. This procedure resulted in the anterior release of the right L5. OLIF51 was followed by extended posterior fixation surgery from L3 to the ilium. Postoperative images showed enlargement of the anterior exit zone of the right L5 nerve root (Figure 2).

The patient was symptom-free at 12 months postoperatively.

Case 2

**Figure 3:**
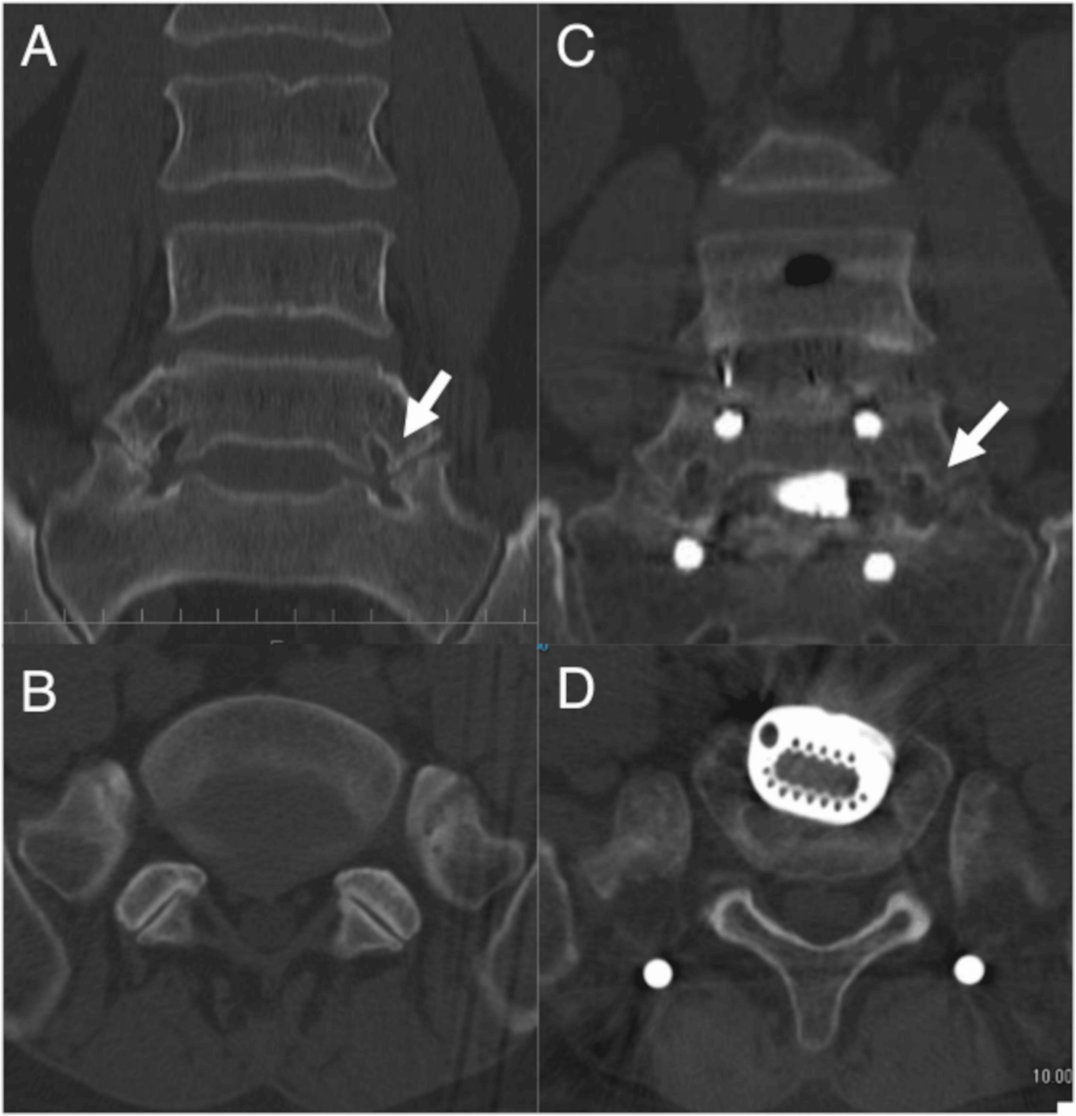
Chronological computed tomography images of Case 2. A (before the procedure), C (after the procedure): Coronal bony CT images. The left L5 nerve root foraminal stenosis (white arrows) is identified and treated with oblique lumbar intervertebral fusion at L5/S1. B (before the procedure), D (after the procedure): Axial images. A large-size artificial intervertebral cage is inserted at the L5/S1 level.

A 46-year-old man presented with pain in the back and lower left extremity due to left L5 neuralgia and underwent lumbar herniation discectomy at L4/L5. Postoperatively, the pain decreased temporarily but increased again. Conservative treatment failed to alleviate the pain. CT images revealed LSTV involvement and the pain was attributed to far-out syndrome (Figure 3A, 3B). As the height of the L5/S1 intervertebral disc had not diminished, a large-size artificial intervertebral cage (degree: 6°, size: 32mm × 25mm × 12mm, SOVEREIGN™ Spinal System; Medtronic plc) was inserted. Additionally, OLIF51 was performed to decompress the anterior exit zone of the left L5 nerve root. The height of the foraminal canal increased (Figure 3C, 3D).

**Figure 4:**
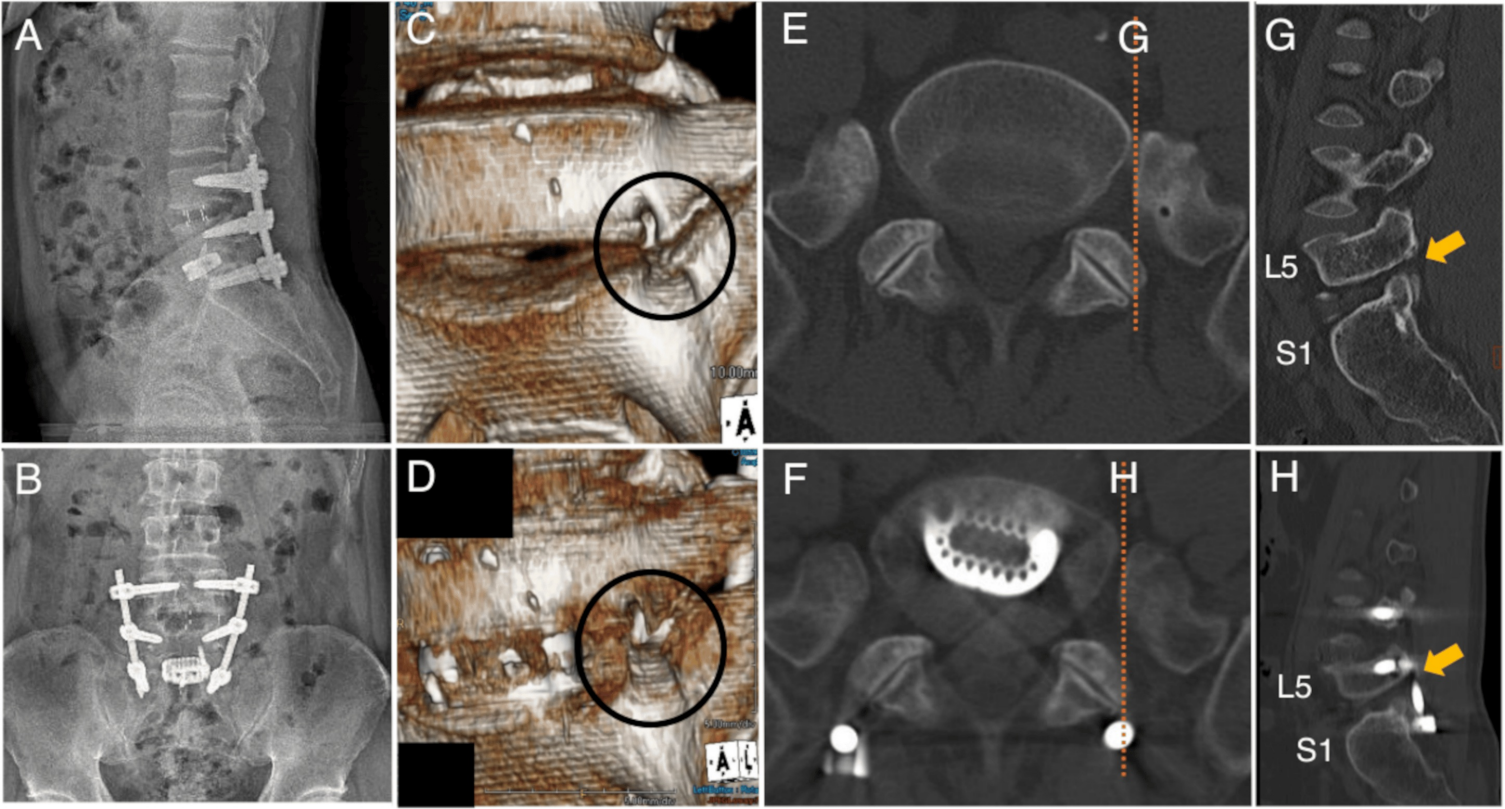
Final radiographic images and chronological computed tomography images of Case 2. A (sagittal image), B (coronal image): Following oblique lumbar intervertebral fusion at L5/S1 (OLIF51), posterior lumbar intervertebral fusion at L4/5 and posterior fixation from L4 to S1 were performed. X-ray images showed intervertebral discs and pedicle screws inserted in posterior fixation surgery from L4 to S1. C, D (three-dimensional reconstructed images): The anterior exit stenosis of the left L5 nerve root is resolved after OLIF51. E (axial CT image), G (sagittal CT image): The L5 nerve root foraminal stenosis was observed preoperatively (orange arrow). F (axial CT image), H (sagittal CT image): The L5 nerve root was posteriorly decompressed along the foraminal canal (orange arrow).

To decompress the L5 nerve root completely, posterior fixation surgery from L4 to S1 was also performed These combined operations successfully resulted in the decompression of the left L5 nerve root along the foraminal canal (Figure 4).

The pain resolved postoperatively. The patient has been followed up at our outpatient clinic and has shown no recurrence for 10 months.

## Discussion

We described two cases of LSTV-related far-out syndrome that were successfully managed with OLIF51. Both patients had undergone operations previously, namely posterior lumbar intervertebral fixation for degenerative lumbar spondylosis in one case and lumbar herniation discectomy in the other. Far-out syndrome was not diagnosed initially in either case owing to the concurrent pathology responsible for L5 neuralgia. OLIF51 effectively resolved the far-out syndrome symptoms in both cases.

Although the far-out syndrome can be managed via the posterior approach, the anterior approach has also been described previously. Abe et al. [6] and Kikuchi et al. [2] reported the treatment of far-out syndrome in a 37-year-old man and a 70-year-old man, respectively, using the extraperitoneal approach. The osteophyte was removed via this approach, and the L5 nerve root was directly decompressed in both cases. However, no description regarding the successful management of far-out syndrome with OLIF51 has been reported.

Based on OLIF performed at the L2-5 level, this approach was also applied to the L5/S1 level because of its advantage in correcting the sagittal and coronal spinal imbalance. Additionally, OLIF51 achieves indirect decompression with spontaneous foraminal enlargement [7,8]. This mechanism can effectively resolve the L5 radiculopathy associated with far-out syndrome. Insufficient anterior zone decompression after the posterior decompression required anterior foraminal enlargement in our first case, while foraminal decompression was necessary at the L5/S1 level in the second case. Particularly, the L5/S1 intervertebral height was relatively preserved in the second case. A large artificial intervertebral disc was inserted at the L5/S1 level after OLIF51, facilitating the L5 foraminal enlargement and resolving far-out syndrome. Although anterior decompression was not achieved enough with posterior decompression surgery in Case 1, the anterior release of the L5 nerve root foramen was successfully performed with OLIF51. Also in Case 2, the increased height of the L5 foramen after OLIF51 resulted in successful indirect decompression of the left L5 nerve root. Combining OLIF51 with posterior fixation surgery, the left L5 nerve root was decompressed enough along the foraminal canal. Therefore, OLIF51 can be a good option for managing this syndrome.

Because of the limited number of far-out syndrome cases managed with OLIF51, further studies comprising more cases with a long-term follow-up are warranted to confirm the treatment effectiveness and establish evidence. The following intraoperative and postoperative complications with OLIF51 have been reported: vascular injury, superior mesenteric artery syndrome, ileus, and L5 radiculopathy [9-11]. Particularly, intraoperative vascular injury can be fatal. Thus, OLIF51 for far-out syndrome should be indicated depending on the surgeon’s experience and skill. Notably, diagnosing LSTV-related far-out syndrome can be challenging when another comorbidity exists. Clinicians should be aware that this rare entity could cause persistent L5 radiculopathy.

## Conclusions

OLIF51 can be an effective surgical option to treat LSTV-related far-out syndrome. As described in our study, this syndrome can coexist with other lumbar degenerative diseases. Clinicians should suspect this rare syndrome in case only posterior decompression or fixation surgery does not resolve L5 neuralgia. OLIF51 anteriorly enlarging the foramen of L5/S1 might resolve L5 neuralgia resulting from LSTV-related far-out syndrome. The indication of OLIF51 for LSTV-related far-out syndrome should be evaluated in the individual case. In case the complete decompression of the L5 nerve root seems warranted, combined OLIF51 and posterior decompression and fixation surgery should also be performed. Further research seems necessary to generalize the indication of OLIF51 for LSTV-related far-out syndrome.
